# Repetitive transcranial magnetic stimulation (rTMS) for obsessive - compulsive disorder (OCD): Naturalistic Observational Study

**DOI:** 10.1192/j.eurpsy.2026.11757

**Published:** 2026-07-31

**Authors:** A. Bolu

**Affiliations:** Private Practice, Ankara, Türkiye

## Abstract

**Introduction:**

Obsessive-compulsive disorder (OCD), with a lifetime prevalence of 1–3%, is a common and disabling psychiatric disorder. Approximately 40–60% of patients with OCD demonstrate resistance to currently available first-line treatments. Repetitive transcranial magnetic stimulation (rTMS) was initially introduced for treatment-resistant depressive disorder, and in subsequent years, its use expanded to treatment-resistant OCD. As evidence on this intervention continues to accumulate, conclusions regarding its therapeutic efficacy will become more definitive.

**Objectives:**

The primary objective of this study was to assess the efficacy of rTMS in patients with treatment-resistant OCD. A secondary aim was to investigate sociodemographic determinants that may serve as predictors of treatment outcomes.

**Methods:**

The present was conducted with a total of 94 patients diagnosed with treatment-resistant OCD. To assess treatment responses, the Yale–Brown Obsessive-Compulsive Scale (Y-BOCS) was administered before and after the rTMS intervention. Based on the rate of change in Y-BOCS scores, an improvement of less than 25% was defined as an insufficient response, 25–50% improvement as a partial response, and more than 50% improvement as a full response. rTMS was delivered using a MagPro R20 device with an MCF-B65 figure-of-eight coil (MagVenture A/S, Denmark). Following informed consent, resting motor thresholds were determined for each patient by an experienced psychiatrist using visual methods. Stimulation was applied over the left dorsolateral prefrontal cortex (DLPFC) at an intensity corresponding to 80–100% of the motor threshold. The protocol consisted of 10 Hz stimulation, delivering 2,400 pulses per session. Treatments were administered once daily on weekdays for a total of 20 sessions across four consecutive weeks.

**Results:**

Of the participants, 54.3% (n = 51) were female and 45.7% (n = 43) were male, with a mean age of 39.6 ± 14.0 years. The average duration of illness was 7.5 ± 7.0 years. A total of 26.6% of participants (n = 25) demonstrated a full response to treatment, whereas 33% (n = 31) were classified as insufficient responders. Partial response was observed in 40.4% of participants (n = 38). No significant association was found between treatment response rates and gender (p > 0.05). However, both duration of illness and patient age were identified as significant factors influencing rTMS treatment response (p ≤ 0.05).

**Conclusions:**

Previous studies have reported variable response rates to rTMS in treatment-resistant OCD In our earlier naturalistic observational study, full response was observed in 21% of patients, while approximately 32% demonstrated a partial response. Potential explanations for these discrepancies include differences in stimulation frequency (low/high), heterogeneity of patient populations, and variation in stimulation targets across studies.

**Disclosure of Interest:**

None Declared

